# Insecticide resistance in *Anopheles arabiensis *(Diptera: Culicidae) from villages in central, northern and south west Ethiopia and detection of *kdr *mutation

**DOI:** 10.1186/1756-3305-3-40

**Published:** 2010-04-25

**Authors:** Meshesha Balkew, Muntaser Ibrahim, Lizette L Koekemoer, Basil D Brooke, Howard Engers, Abraham Aseffa, Teshome Gebre-Michael, Ibrahim Elhassen

**Affiliations:** 1 Aklilu Lemma Institute of Pathobiology, Addis Ababa University, Addis Ababa, Ethiopia; 2 Institute of Endemic Diseases, University of Khartoum, Khartoum, Sudan; 3 Vector Control Reference Unit, National Institute for Communicable Diseases of the NHLS, Private Bag X4, Sandringham, Johannesburg 2131, South Africa; 4 Division of Virology and Communicable Disease Surveillance, School of Pathology of the University of the Witwatersrand and the National Health Laboratory Service, Johannesburg, South Africa; 5 Armauer Hansen Research Institute, Addis Ababa, Ethiopia

## Abstract

**Background:**

*Anopheles arabiensis *is the major vector of malaria in Ethiopia. Malaria vector control in Ethiopia is based on selective indoor residual spraying using DDT, distribution of long lasting insecticide treated nets and environmental management of larval breeding habitats. DDT and pyrethroid insecticides are neurotoxins and have a similar mode of action on the sodium ion channel of insects. It was therefore necessary to verify the insecticide susceptibility status of *An. arabiensis*, to better understand the status of cross-resistance between DDT and the pyrethroids in this species as well as to detect a resistant gene.

**Methods:**

Standard WHO insecticide susceptibility tests were conducted on adults reared from larval and pupal collections from breeding sites at three villages namely: Sodere in the Rift Valley, Gorgora in the north and Ghibe River Valley in the south west of Ethiopia. The occurrence of cross-resistance between pyrethroids and DDT was determined using a DDT selected laboratory colony originally collected from Gorgora. Phenotypically characterized mosquitoes were tested for the presence of knockdown resistance (*kdr*) alleles using the standard polymerase chain reaction assay.

**Results:**

All *An. gambiae *s.l. specimens assayed by PCR were identified as *An. arabiensis*. The knockdown and mortality results showed *An. arabiensis *resistance to DDT in all villages, resistance to deltamethrin and permethrin in the Ghibe River Valley and permethrin resistance in Gorgora. Bioassay susceptibility tests also indicated the presence of cross-resistance between DDT and permethrin, but not between DDT and deltamethrin. The knockdown resistance *(kdr) *mutation of leucine to phenylalanine in the sodium ion channel gene was detected in populations from Gorgora and the Ghibe River Valley.

**Conclusion:**

Since *An. arabiensis *shows high levels of resistance to DDT in all villages tested and varying pyrethroid resistance in Gorgora and the Ghibe River valley, precautionary measures should be taken in future vector control operations. Moreover, the status of resistance in other locations in Ethiopia and the spread of resistant gene (s) should be investigated.

## Background

Malaria vector control in Ethiopia is targeted mainly against *Anopheles arabiensis *as this species is responsible for transmitting *Plasmodium falciparum *and *P. vivax *[1]. Vector control measures including selective indoor residual spraying of dichlorodiphenyltrichloroethane (DDT), distribution of long lasting insecticide treated mosquito nets (LLINs) and source reduction of larval habitats are currently implemented by the Federal Ministry of Health in collaboration with international and non-governmental organizations. The history of utilization of DDT in the country dates back to the mid 1950s with small scale trials followed by wide and extensive application during the malaria eradication period. Thus, DDT has been in use for more than four decades. The emergence of DDT resistance in *An. arabiensis *has been reported from different localities in Ethiopia [2,3] and the highest levels of resistance were recorded from ArbaMinch in the South and Gambella in the West [2]. DDT resistance has also been demonstrated in populations of *An. pharoensis *in two localities [4,5], one of three secondary malaria vectors of Ethiopia (the other two being *An. funestus *and *An. nili*)[1].

DDT and pyrethroid insecticides are insect neurotoxins that interfere with ion flow regulation across the sodium ion channels. Ion channel modification via an amino-acid substitution leads to reduced target site sensitivity known as knockdown resistance (*kdr*). The substitution of leucine at position 1014 for either a phenylalanine or a serine has been reported in *An. gambiae *s.s. from West and East Africa, respectively [6,7]. These mutations have also been reported in *An. arabiensis *[8,9].

In view of the prolonged utilization of DDT for vector control, the application of different agricultural pesticides in agrodevelopment areas and the planned up-scaling of pyrethroid treated LLINs distributionace in the areasurrounding areas is found in Metehara. ArbaMinch is Metehara is one of the, it was necessary to evaluate the insecticide susceptibility status and genetic basis of insecticide resistance and cross-resistance in *An. arabiensis *from three selected localities in Ethiopia.

## Methods

### Study localities

The study was conducted at three localities namely Ghibe River Valley in the west (hereafter referred to as Ghibe), Sodere hot springs in the Rift Valley and Gorgora in the North West where DDT spraying has been practiced for a long time. Ghibe (8°14'N, 37°35'E, altitude 1,040-1,080 m) is situated 185 kms south-west of Addis Ababa. It has a long history of state owned farming in which vegetables, maize and citrus fruits are produced. The International Livestock Research Institute (ILRI) used to conduct research studies on cattle trypanosomiasis and *Glossina *in the same locality as well as in Tolay (Upper Ghibe River Valley). To control tsetse flies, the ILRI introduced deltamethrin (pyrethroid) treated cattle as well as targets impregnated with the same insecticide [10]. Currently, the Institute provides technical support to local farmers at both study sites for the application of cyfluthrin (pyrethroid) on cattle (Dr. Woudyalew Mulat, personal communication). Sodere hot springs (8° 24'N and 39° 23'E, altitude 1,360 m) is located in the middle course of the Rift Valley about 125 kms east of Addis Ababa. Gorgora (12° 12'N and 37° 16' E, altitude 1,800 m) is located at the northern tip of Lake Tana, about 800 kms north of Addis Ababa. Three private agricultural developments are underway near Gorgora.

### Insecticide susceptibility tests

Insecticide susceptibility tests were carried out at different times between November 2005 and April 2007. Mosquito larvae and pupae were collected from various breeding sites and reared to adults. Two to three days old, non blood fed adult females morphologically identified as *An. gambiae *s. l were exposed to discriminating dosages of 4% DDT, 0.05% deltamethrin and 0.75% permethrin using WHO papers (obtained from Vector Control Research Unit, School of Biological Sciences, Universiti Sains Malaysia) and bioassay tubes for 1 hour [11]. Number of mosquitoes knocked down during exposure was recorded at intervals of 5 minutes and the proportions of survivors and dead mosquitoes were recorded 24 hours post exposure. The same number of mosquitoes was exposed to insecticide free papers as controls.

Samples of dead and surviving mosquitoes were preserved in 95% ethanol and kept in a freezer (-21°C) for subsequent molecular species identification and *kdr *analysis. The quality of impregnated papers was checked on a laboratory colony of *An. arabiensis *which is susceptible (100% mortality) to DDT, permethrin and deltamethrin. The colony is maintained at the Aklilu Lemma Institute of Pathobiology, Addis Ababa University, since September 2001 from original collection of mosquitoes from Debrezeit, 45 kilometers east of Addis Ababa.

### Species identification

A polymerase chain reaction (PCR) assay was carried out to differentiate *An. arabiensis *from its *An. gambiae *complex sibling species according to the method of Scott *et al*. [12].

### Knockdown resistance gene detection

For detection of *kdr *mutations, genomic DNA was isolated from 70 mosquitoes according to the method of Collins *et al. *[13]. Two separate PCR reactions were run, one to detect alleles of the leucine-phenylalanine substitution, the other, to detect wild-type susceptible alleles following the methods described in Matambo *et al*. [14] and Abdalla *et al*. [15]. The occurrence of *kdr *was confirmed by direct sequencing of the 293 base pair fragment of the sodium channel gene amplified using Agd1 and Agd2 primers [6]. Amplicons were submitted to Inqaba Biotec Company, Pretoria, South Africa for sequencing in both directions.

### Selection for resistance, colony maintenance and cross resistance evaluation

*An. arabiensis *strain originating from a collection of wild females from Gorgora was selected for resistance to DDT. Selections were carried out in the insectary at the Aklilu Lemma Institute of Pathobiology by exposing male and female adults over generation to 4% DDT impregnated papers. Full resistance to DDT (zero mortality) was achieved in the 6^th ^generation. Subsequently insecticide susceptibility tests using 0.75% permethrin and 0.5% deltamethrin impregnated papers were carried out on the 8^th ^generation according to the standard WHO method [11]. Tests included 5 replicates of 20 unfed, 2-3 day old adult females per replicate. Controls included exposure of females from the same cohort to insecticide free papers.

### Data analysis

Percentage mortalities 24 h post exposure was used to assess the status of susceptibility/resistance to insecticides. The KT_50 _and KT_90 _(time to knockdown 50% and 90% mosquitoes, respectively) values were calculated for each insecticide using probit analysis in SPSS 15.0 for windows. Fisher's exact test was applied to observe associations between genotypes and phenotypes. Chi-square statistics was used to analyze allelic and genotype frequencies.

## Results

### Mosquito species identification

300 *An. gambiae *s.l. samples were assayed by PCR. All were identified as *An. arabiensis*.

### Insecticide susceptibility test results

Exposure to DDT induced significantly reduced mortality in *An. arabiensis *from all study localities, implicating resistance according to established criteria [11]. The highest resistance was recorded from Ghibe followed by Gorgora and Sodere (Table 1). Mortality due to permethrin (85%) and deltamethrin (80.6%) exposure in samples from Gorgora is strongly suggestive of pyrethroid resistance. The KT_50 _and KT_90 _values are also elevated. *Anopheles arabiensis *resistance to pyrethroids has only progressed to a high level in Ghibe.

**Table 1:** Mortality and knockdown effect of insecticides on *An. arabiensis *from three localities in Ethiopia.

Locality	Insecticide	Number tested	Percent mortality	KT_**50 **_in minutes (95% confidence interval)	KT_**90 **_in minutes (95% confidence interval)
Gorgora	DDT	198	49.5	*	*
	Permethrin	140	85.0	19.6 (17.5-21.6)	28.7(26.2-32.5)
	Deltamethrin	160	80.6	25.3 (23.2-27.5)	35.9(33.1-40.0)
Ghibe	DDT	160	3.8	**	**
	Permethrin	60	31.7	***	
	Deltamethrin	160	31.3	37.6 (34.7-40.5)	56.6(52.8-61.7)
Sodere	DDT	118	78.8	****	****
	Permethrin	80	100	17.8 (15.9-19.7)	25.8(23.4-25.9)
	Deltamethrin	120	99.2	21.9 (19.9-23.9)	31.0(28.6-34.8)

*60% were knockeddown at the end of 80 m, ** only 4 mosquitoes were knocked down at the end of 80 minutes,***41.7% were knockeddown at the end of 80 m**** 77.1% were knockeddown at the end of 80 m.

### *kdr *gene detection

Thirty two *An. arabiensis *specimens from Gorgora and 38 from Ghibe were examined for the occurrence of 1014 F *kdr *mutation [6]. The frequency of genotypes of tested mosquitoes on each insecticide is separately depicted in Table 2. The allelic and genotypic frequencies are in Hardy-Weinberg equilibrium (Gorgora *X*^2 ^= 0.02, p = 0.99; Ghibe *X*^2 ^= 2.62, p > 0.27) (Table 3). The genotype frequency of *An. arabiensis *from Gorgora is statistically different from the population of Ghibe (Fisher's exact test, p < 0.05).

**Table 2:** Genotypic frequency of *kdr *from bioassayed *An. arabiensis *from Gorgora and Ghibe, Ethiopia

Locality	Insecticide	Phenotypes	Number	Genotype (%)		
				SS	RS	RR
Gorgora	DDT	Susceptible	2	2 (100)	0	0
		Resistant	12	1 (8.3)	8 (66.7)	3 (25.0)
	Permethrin	Susceptible	7	5 (71.4)	1 (14.3)	1 (14.3)
		Resistant	11	4 (36.4)	7 (63.6)	0
Ghibe	DDT	Susceptible	5	0	4 (80.0)	1 (20.0)
		Resistant	9	0	6 (66.7)	3 (33.3)
	Permethrin	Susceptible	7	0	3 (42.9)	4 (57.1)
		Resistant	3	0	0	3 (100)
	Deltamethrin	Susceptible	6	0	2 (33.3)	4 (66.7)
		Resistant	8	0	2 (25.0)	6 (75.0)

SS = homozygote susceptible, RS = heterozygote resistant, = RR homozygous resistant

**Table 3:** Comparison of genotype determination between PCR and sequencing of Sodium channel gene of *An. arabiensis *from Gorgora and Ghibe River Valley, Ethiopia.

Locality	Number sequenced	PCR results	Sequence results
Gorgora	7	3 RS	Same as PCR
		4 SS	
Ghibe	7	4 RR	5 RR
		3 RS	2 RS

The occurrence of *kdr *mutations were further confirmed by sequencing the 293 base-pair fragment of the sodium channel gene of 14 randomly chosen *An. arabiensis *from Gorgora and Ghibe. The sequences revealed similar results (with only one exception from Ghibe) with that of PCR confirming the presence of 1014F *kdr *gene (Table 3).

### Cross-resistance tests

Cross-resistance tests on F_8 _DDT resistant *An. arabiensis *from Gorgora against permethrin revealed lower susceptibility with 52% mortality (Table 4). A similar proportion was knocked down at the end of 80 minutes. In contrast, deltamethrin caused 98% mortality. The KT_50 _was 29.3 minutes which was slightly higher than the KT_50 _of the non-selected vector population (25.3 minutes).

**Table 4:** Bioassay test results of base and DDT selected F_8 _*An. arabiensis *from Gorgora.

Insecticide	Number tested	% mortality	**KT**_**50**_**(minutes)**	% mortality in base	**KT**_**50**_**(minutes) in base**
Permethrin	100	52.0*	No. KD	85.0	19.6 (17.5-21.6)
Deltamethrin	100	98.0	29.3 (27.0-31.5)	80.6	25.3 (23.2-27.5)

No. KD = only 56% were knocked down * Control mortality was less than 5%.

## Discussion

The insecticide susceptibility data indicate high levels of resistance to DDT in *An. arabiensis *at all localities, although varying level of mortality and knockdown were observed. The highest level of resistance as shown by the survival rates to DDT was recorded in Ghibe (96%) followed by Gorgora (50.5%) and Sodere (21.2%). Resistance to DDT is higher than previously reported in *An. arabiensis *from Gambela (76%) in the West and ArbaMinch (60%) in the South [2]. The results obtained from Gorgora are comparable to those of the eastern localities (Metehara and MelkaWorer) where 45-50% resistance was reported [3]. Ameneshewa [16] noted 30% resistance from Gergedi which is close to Sodere.

*Anopheles arabiensis *from Ghibe shows high resistance (about 70%) to permethrin and deltamethrin as well. The Gorgora population also shows signs of resistance to pyrethroids (15%-20%) based on final mortality data. The time to 50% knockdown at Gorgora was higher (KT_50 _= 19.6 minutes for permethrin and 25.3 minutes for deltamethrin) than that of a susceptible laboratory colony (KT_50_s of 14.6 and 18.3 minutes for permethrin and deltamethrin, respectively: data not shown) as well as of a susceptible reference strain of *An. gambiae *[17,18]. The pyrethroid resistance levels in Gorgora at the present are unlikely to pose an epidemiological threat but routine periodic monitoring to determine levels of resistance are needed. *Anopheles arabiensis *from Sodere are susceptible to the two pyrethroids and yet, their knockdown times are similar to those recorded in Gorgora. In neighboring Sudan, *An. arabiensis *showed comparable levels of resistance to permethrin (final mortalities varying between 10-55% in Gezira and 6-22% in Sennar) [15]. However, populations of this species proved susceptible to pyrethroids at three localities in the eastern part of the country [9]. Variable levels of resistance to DDT and pyrethroids in *An. arabiensis *populations in neighboring countries raises concerns because of unrestricted gene flow between populations through migration.

The West African *kdr *mutation was detected in *An. arabiensis *from Ghibe and Gorgora. Resistance to pyrethroids and DDT in *An. gambiae *associates closely with *kdr *[6,7,19,20]. However, a similar correlation of *kdr *with the phenotypic expression of resistance could neither be established in a DDT-selected *An. arabiensis *laboratory strain from Sudan [14] nor in natural populations [9,15]. Matambo *et al*. [14] and Abdalla *et al*. [15] observed discrepancies in their PCR data compared to sequenced samples. The findings from Sudan samples also showed that *kdr *is found in phenotypically insecticide susceptible mosquitoes, not being restricted to resistant individuals alone. The data from Gorgora and Ghibe show the same phenomenon whereby *kdr *homozygotes were recorded in samples characterized as phenotypically susceptible to insecticides. Brooke [21] argues that *kdr *might not be the only mechanism that confers resistance to DDT and pyrethroids and suggests the potential involvement of enzyme detoxification. Target site insensitivity coupled with enzyme detoxification has been described in *An. gambiae *and *Culex quinquefasciatus *from Benin [22].

The insecticide resistance in *An. arabiensis *from Ghibe and Gorgora and reduced level from Sodere may be attributable to differential insecticide selection pressure. Applications of insecticide against agricultural pests and disease vectors including anophelines and tsetse flies are practiced intensively in Ghibe and, to a lesser extent, in Gorgora. Documented information is lacking on the types of insecticides in use at these agricultural localities except that deltamethrin and cyflumethrin pour-ons are used on cattle in Ghibe [10]. In Gorgora, different types of insecticides have been in use for a number of years to control agricultural pests (Ato Teklu, personal communication), although it was difficult to establish whether DDT has been used. Use of agricultural pesticides in Ghibe may account for the increased resistance of *An. arabiensis *to deltamethrin and permethrin. Pyrethroids have not been used for malaria vector control in these areas until the recent distribution of LLINs which started in 2005. Agricultural pesticides may exert selective pressure on malaria vectors by leaching into breeding habitats so that the immature stages are continuously exposed. In West Africa, DDT application on farms during the early WHO malaria vector control era and extensive use of pyrethroids on cotton farms has been attributed to increased resistance of *An. gambiae *to pyrethroids as well as increased frequency of the *kdr *genotype in many countries [20,22,23]. In urban areas, insecticides for households and the expansion of urban agriculture are suggested to cause resistance in malaria vectors in West Africa [24].

Cross-resistance between DDT and permethrin was shown in a DDT-resistant laboratory colony of *An. arabiensis *originating from Gorgora possibly caused by *kdr*. By contrast, cross resistance between DDT and deltamethrin was not recorded in *An. arabiensis *samples from Sudan [14]. In this case the influence of *kdr *might be less important because the test population carrying the *kdr *allele showed a high level of susceptibility to deltamethrin, suggesting the existence of an independent DDT resistance mechanism. The absence of cross-resistance between DDT and deltamethrin in Gorgora DDT-selected *An. arabiensis *is encouraging in terms of LLINs utilization, although the moderate deltamethrin resistance recorded requires careful monitoring and consideration.

The potential impact of resistance on LNs use and indoor spraying with DDT in Ghibe is unknown and needs to be established. Permethrin resistance in *An. arabiensis *from Sodere is lower than in Ghibe, and regular monitoring should identify any changes in the level of resistance in association with the continued use of LLINs. In Kenya, lower susceptibility of *An. gambiae *to permethrin was indicated in villages where permethrin impregnated mosquito nets were implemented as compared to villages without nets in a period of one year and the reduction was linked to increased detoxification caused by elevated cytochrome P450 monooxygenase activity together with *kdr-w*[25]. Similarly, another study by Stump *et al*. [26] confirmed an increased frequency of *kdr *in villages with nets.

In conclusion, since the country relies on DDT for indoor residual sprays, the development of resistant populations of *An. arabiensis *implies careful consideration and monitoring of ongoing vector control program. There is also a need to monitor pyrethroid susceptibility in wider areas covering a number of vector populations.

## Competing interests

The authors declare that they have no competing interests.

## Authors' contributions

MB conducted the field and laboratory study and drafted the manuscript, IE and MI involved in project design and conducted the study, LLK and BDB facilitated the molecular laboratory study and commented on the manuscript, HE contributed to project design, facilitated the supply of WHO bioassay materials and reviewed the draft manuscript, AA and TGM facilitated the laboratory and field study and participated in drafting the manuscript. All co-authors have read the manuscript.

## References

[B1] Gebreyesus AT Deressa W Witten HK Getachew A Seboxa T Berhane Y, Haile Mariam D, Kloos H Malaria Epidemiology and Ecology of Health and Disease in Ethiopia 2006 Shama Books. Addis Ababa

[B2] Abose T Yeebiyo Y Olana D Alamirew D Beyene YA Regassa L Mengesha A Re-orientation and Definition of the Role of Malaria Vector Control in Ethiopia: The Epidemiology and Control of Malaria with Special Emphasis on the Distribution, Behaviour and Susceptibility of Insecticides of Anopheline Vectors and Chloroquine Resistance in Zwai, Central Ethiopia and Other Areas 1998 World Health Organization WHO/Mal/1998.1085

[B3] Balkew M Gebre-Michael T Hailu A Insecticide susceptibility of *Anopheles arabiensis *in two agrodevelopment localities in eastern Ethiopia Parasssitologia 2003 45 1 3 15270536

[B4] Wezam A Seulu F *Anopheles pharoensis *susceptibility status to DDT and malathion in Zwai area, south-central Ethiopia Ethiop Pharm J 1994 12 45 50

[B5] Balkew M Elhassen I Ibrahim M Gebre-Michael T Engers H Very high DDT-resistant population of *Anopheles pharoensis *Theobald (Diptera: Culicidae) from Gorgora, Northern Ethiopia Parasite 2006 13 327 329 17285855 10.1051/parasite/2006134327

[B6] Martinez-Torres D Chandre F Williamson MS Darriet F Berge JB Devonshire AL Guillet P Pasteur N Pauron D Molecular characterization of pyrethroid knockdown resistance *(kdr*) in the major malaria vector *Anopheles gambiae *s.s Insect Mol Biol 1998 7 179 184 10.1046/j.1365-2583.1998.72062.x 9535162

[B7] Ranson H Jansen B Vulule JM Wang X Hemingway J Collins FH Identification of a point mutation in the voltage-gated sodium channel gene of Kenyan *Anopheles gambiae *associated with resistance to DDT and pyrethroids Insect Mol Biol 2000 9 491 497 10.1046/j.1365-2583.2000.00209.x 11029667

[B8] Verhaeghen K Van Bortel W Roelants P Backeljau T Coosemans M Detection of the East and West African *kdr *mutation in *Anopheles gambiae *and *Anopheles arabiensis *from Uganda using a new assay based on FRET/Melt Curve analysis Malaria J 2006 5 16 10.1186/1475-2875-5-16 PMC1409791 16504072

[B9] Himeidan YE Dukeen MY El-Rayah el A Adam I *Anopheles arabiensis: *abundance and insecticide resistance in an irrigated area of eastern Sudan East Med Hlth J 2004 10 167 74 16201723

[B10] Rowlands GJ Leak SG Mulatu W Nagda SM Wilson A d'Ieteren GD Use of deltamethrin 'pour-on' insecticide for the control of cattle trypanosomosis in the presence of high tsetse invasion Med Vet Entomol 2001 15 87 96 10.1046/j.1365-2915.2001.00272.x 11297107

[B11] WHO Test procedures for insecticide resistance monitoring in malaria vectors, bio-efficacy and persistence of insecticides on treated surfaces 1998 World Health Organization WHO/CDS/MAL/98.12

[B12] Scott JA Brogdon WG Collins FH Identification of single specimens of the *Anopheles gambiae *complex by the polymerase chain reaction Am J Trop Med Hyg 1993 49 520 529 8214283 10.4269/ajtmh.1993.49.520

[B13] Collins FH Mendez MA Rasmussen MO Mehaffey PC Besansky NJ Finnerty V A ribosomal RNA gene probe differentiates member species of the *Anopheles gambiae *complex Am J Trop Med Hyg 1987 37 37 41 2886070 10.4269/ajtmh.1987.37.37

[B14] Matambo TS Abdalla H Brooke BD Koekemoer LL Mnzava A Hunt RH Coetzee M Insecticide resistance in the malarial mosquito *Anopheles arabiensis *and association with the *kdr *mutation Med Vet Entomol 2007 21 97 102 10.1111/j.1365-2915.2007.00671.x 17373952

[B15] Abdalla H Matambo TS Koekemoer LL Mnzava AP Hunt RH Coetzee M Insecticide susceptibility and vector status of natural populations of *Anopheles arabiensis *from Sudan Trans R Soc Trop Med Hyg 2008 102 263 71 10.1016/j.trstmh.2007.10.008 18054056

[B16] Ameneshewa B The behaviour and biology of *Anopheles arabiensis *in relation to the epidemiology and control of malaria in Ethiopia PhD thesis 1995 University of Liverpool

[B17] Chandre F Darriet F Duchon S Finot L Manguin S Carnevale P Guillet P Modifications of pyrethroid effects associated with *kdr *mutation in *Anopheles gambiae* Med Vet Entomol 2000 14 81 8 10.1046/j.1365-2915.2000.00212.x 10759316

[B18] Etang J Manga L Chandre F Guillet P Fondjo E Mimpfoundi R Toto JC Fontenille D Insecticide susceptibility status of *Anopheles gambiae *s.l (Diptera: Culicidae) in the Republic of Cameroon J Med Entomol 2003 40 491 7 10.1603/0022-2585-40.4.491 14680116

[B19] Chandre F Darrier F Manga L Akogbeto M Faye O Mouchet J Guillet P Status of pyrethroid resistance in *Anopheles gambiae *sensu lato Bull World Health Organ 1999 77 230 234 10212513 PMC2557627

[B20] Diabaté A Brengues C Baldet T Dabire KR Hougard JM Akogbeto M Kengne P Simard F Guillet P Hemingway J Chandre F The spread of the Leu-Phe *kdr *mutation through *Anopheles gambiae *complex in Burkina Faso: genetic introgression and *de novo *phenomena Trop Med Int Hlth 2004 9 1267 1273 10.1111/j.1365-3156.2004.01336.x 15598258

[B21] Brooke BD *kdr *: can a single mutation produce an entire insecticide resistance phenotype? Trans R Soc Trop Med Hyg 2008 102 524 5 10.1016/j.trstmh.2008.01.001 18295809

[B22] Corbel V N'Guessan R Brengues C Chandre F Djogbenou L Martin T Akogbeto M Hougard JM Rowland M Multiple insecticide resistance mechanisms in *Anopheles gambiae*. and *Culex quinquefasciatus *from Benin, West Africa Acta Tropica 2007 101 207 16 10.1016/j.actatropica.2007.01.005 17359927

[B23] Müller P Chouaibou M Pignatelli P Etang J Walker ED Donnelly MJ Simard F Ranson H Pyrethroid tolerance is associated with elevated expression of antioxidants and agricultural practice in *Anopheles arabiensis *sampled from an area of cotton fields in Northern Cameroon Mol Ecol 2008 17 1145 55 10.1111/j.1365-294X.2007.03617.x 18179425

[B24] Elissa N Mouchet J Riviere F Meunier JY Yao K Resistance of *Anopheles gambiae *s.s to pyrethroids in Cote d'Ivoire Ann Soc Belge Med Trop 1993 73 291 4 8129474

[B25] Vulule JM Beach RF Atieli FK McAllister JC Brogdon WG Roberts JM Mwangi RW Hawley WA Elevated oxidase and esterase levels associated with permethrin tolerance in *Anopheles gambiae *from Kenyan villages using permethrin-impregnated nets Med Vet Entomol 1999 13 239 44 10.1046/j.1365-2915.1999.00177.x 10514048

[B26] Stump AD Atieli FK Vulule JM Besansky NJ Dynamics of the pyrethroid knockdown resistance allele in western Kenyan populations of *Anopheles gambiae *in response to insecticide-treated bed net trials Am J Tropl Med Hyg 2004 70 591 6 15210997

